# Development and Testing of a Mobile Phone App for Self-Monitoring of Calcium Intake in Young Women

**DOI:** 10.2196/mhealth.5717

**Published:** 2017-03-07

**Authors:** Ilona Tay, Suzanne Garland, Alexandra Gorelik, John Dennis Wark

**Affiliations:** ^1^ Department of Medicine University of Melbourne Parkville, VIC Australia; ^2^ The Royal Women's Hospital Department of Microbiology & Infectious Diseases Parkville, VIC Australia; ^3^ Department of Obstetrics & Gynaecology University of Melbourne Parkville Australia; ^4^ Murdoch Childrens Hospital Parkville Australia; ^5^ Royal Melbourne Hospital Melbourne EpiCentre University of Melbourne Parkville, VIC Australia; ^6^ Royal Melbourne Hospital Bone and Mineral Medicine Parkville, VIC Australia

**Keywords:** behavior therapy, cell phones, health behavior, primary prevention, self care, telemedicine

## Abstract

**Background:**

Interventions to prevent osteoporosis by increasing dairy intake or physical activity in young women have been limited to increasing osteoporosis knowledge and awareness. However, findings have shown that this does not always lead to a change in behaviors. Self-monitoring using mobile devices in behavioral interventions has yielded significant and positive outcomes. Yet, to our knowledge, mobile self-monitoring has not been used as an intervention strategy to increase calcium intake, particularly in young women, for better bone health outcomes.

**Objective:**

As development and testing of mobile app–based interventions requires a sequence of steps, our study focused on testing the acceptability and usability of Calci-app, a dietary app to self-monitor calcium consumption, before it is used in a behavioral change intervention in young women aged 18-25 years.

**Methods:**

Calci-app development followed 4 steps: (1) conceptualization, (2) development and pretesting, (3) pilot testing, and (4) mixed methods evaluation.

**Results:**

We present the development process of Calci-app and evaluation of the acceptability and usability of the app in young women. Overall, 78% (31/40) of study participants completed the 5-day food record with high compliance levels (defined as more than 3 days of full or partial completion). There was a significant reduction in the proportion of participants completing all meal entries over the 5 days (*P*=.01). Participants generally found Calci-app easy and convenient to use, but it was time-consuming and they expressed a lack of motivation to use the app.

**Conclusions:**

We present a detailed description of the development process of Calci-app and an evaluation of its usability and acceptability to self-monitor dietary calcium intake. The findings from this preliminary study demonstrated acceptable use of Calci-app to self-monitor calcium consumption. However, for regular and long-term use the self-monitoring function in Calci-app could be expanded to allow participants to view their total daily calcium intake compared with the recommended daily intake. Additionally, to facilitate sustainable lifestyle behavior modifications, a combination of various behavior change techniques should be considered, such as education, goal setting, and advice to participants based on their stage of change. The feedback on barriers and facilitators from testing Calci-app will be used to design a bone health mHealth intervention to modify risky lifestyle behaviors in young women for better bone health outcomes.

## Introduction

Osteoporosis is an outcome of poor bone health and manifests as porous bone with low bone density and poor bone strength. It was estimated that 4.74 million Australians older than 50 years had osteoporosis or osteopenia in 2012 [1]. These conditions affect more women than men over the age of 55 years [2]. Without intervention, the prevalence of osteoporosis is expected to increase by 31% by 2022 because of population aging [2]. This condition is costly, accounting for Aus $2.75 billion in total costs in 2012 in Australia [1], and it causes significant morbidity and mortality [3].

The conceptualization phase of this study was prompted by the high prevalence and socioeconomic burden of osteoporosis. Emphasis should be placed on preventing this condition by maximizing peak bone mass in young women before bone accrual stops in approximately the third decade of life [4]. During the transitional phase of life (18-24 years), young adults are more likely to practice risky lifestyle behaviors and cultivate bad lifestyle habits that may have deleterious effects on future bone health [5]. Yet young adults tend to be underrepresented in medical and population health research, being highly mobile and more challenging to recruit and retain than both older and younger populations [6]. Nevertheless, this phase of life is viewed as a window of opportunity for the formation of healthy lifestyle habits in order to avoid suboptimal acquisition and maintenance of peak bone mass and, hence, poor bone health in later life.

Interventions to prevent osteoporosis by increasing dairy intake or physical activity in young women have been limited to increasing osteoporosis knowledge and awareness. However, research has shown that this approach does not always lead to a change in relevant behaviors [7-11]. This prompts a call for other intervention methods. Self-monitoring using mobile devices in behavioral interventions has yielded significant and positive outcomes such as weight loss [12], better blood glucose control in patients with type 2 diabetes mellitus [13], and improved depressive symptoms in young people with mild depression [14]. Yet, to our knowledge, mobile self-monitoring has not been used as an intervention strategy to maximize peak bone mass acquisition for better bone health, particularly in young women.

Numerous lifestyle factors contribute to the attainment of optimal peak bone mass, such as physical activity [15], avoidance of smoking [16], and calcium intake [17]. In Australia, less than 40% of women aged 19-50 years have calcium intakes that meet the estimated average requirement of 840 mg/day [18]. Indeed, the typical average calcium intake in young women aged 15-30 years is only 60% of the estimated average requirement [19]. Osteoporosis is a multifactorial disease; therefore, behavior change interventions for better bone health should take into account various lifestyle factors. As development of mobile app–based interventions requires a sequence of steps [20], this study focused on testing one of the apps, *Calci-app*, that we intend to use in future mHealth behavior change interventions. There are commercially available mobile phone apps such as MyFitnessPal and Easy Diet Diary for tracking one’s diet and reporting of nutrient intake [21]. However, they emphasize macronutrient intake, particularly caloric intake. At the time when this study was carried out, calcium content was displayed in MyFitnessPal as a total percentage consumed against the daily requirement and it included an American food database. Although Easy Diet Diary used an Australian food database, it did not display calcium intake. Therefore, we developed Calci-app specifically for this study to report the actual calcium levels in food and beverages that are typical of an Australian diet.

Self-monitoring dietary intake involves the recording of the type and amount of food and beverages consumed and this is traditionally done on paper [22]. One of the drawbacks is the burden placed on participants to write down detailed dietary information with paper and pen. With recent technological advances, the burden of self-monitoring on paper diaries has reduced with the use of mobile devices such as mobile phone apps, websites [23], and personal digital assistants (PDAs) [24]. About 77% of Australians carry their mobile phones with them every day [3], and this widespread use of mobile phones can be harnessed for intervention delivery. Research has shown that young people prefer self-monitoring dietary intake on computers and mobile phones over paper [25], and these methods have similar accuracy.

Therefore, our study aimed to assess the usability and acceptability of Calci-app in young women to self-monitor dietary calcium intake and its potential for use in a bone health mHealth behavior change intervention. It should be noted that Calci-app was designed as a monitoring tool for calcium intake, rather than a behavior change intervention in itself. Its functions are consistent with this purpose. It will enable various forms of feedback to users to be added in the implementation of future interventions.

## Methods

### Initial Development

Calci-app is part of a large observational health study, the Young Female Health Initiative (YFHI), which is being conducted in young women aged 16-25 years living in the state of Victoria, Australia. YFHI is the most comprehensive study yet undertaken to examine young women’s health in Australia across many different health domains. YFHI combines Web-based and remote data collection methods with extensive site visits to collect detailed biodata for a wide range of health measures to examine the interplay between the participants’ lifestyles, behaviors, physical health, and mental health [26].

Calci-app was developed specifically for this study in collaboration with Nowpos M-Solutions Pvt Ltd (Hyderabad, India). It was developed for both Apple iPhones and Android phones. Development of Calci-app was completed through an iterative process over a period of 16 weeks in 3 phases: (1) initial development, (2) beta testing, and (3) pilot testing. During this period, the research team held weekly progress meetings with Nowpos developers.

In the first phase, Nowpos was provided with (1) a food database [27], (2) app objectives, and (3) a description of expected app graphics, functionalities, and navigation. The main features included in the initial development were (1) food categories, (2) portion size entries, (3) a 48-hour data entry window period, (4) manual submission of completed food entries, and (5) 3 times/day automatic reminders.

The food database was taken from AUSNUT 2007 (Food Standards Australia New Zealand), which contained more than 3800 items. In an initial attempt to simplify food entries for the participants, we sorted the items into 12 categories, such as meat, seafood and fish, and fruit and vegetable. However, as it might create frustration in users having to determine the correct categories, we decided to remove the food categories. Instead, we added a search function for participants to enter keywords that would pull out relevant food items from the database. Portion sizes were automatically entered according to the standard servings provided in AUSNUT 2007, and the quantity could be adjusted by participants using their phones’ type pad and the measurement unit could be selected via a drop-down list. Participants were able to view the calcium content next to each of the food or beverage items that they added. In order to minimize recall bias, we set up a window period of 48 hours for data entry, in which entries were restricted to the actual scheduled day plus the following day. After entering their data, participants were required to click on a “submit” button to confirm and send their entries to the backend database.

If no food or drink were consumed, users were asked to click on a button indicating that nothing had been consumed for a particular mealtime. This process was designed to prevent the app from mistaking the blank meal entry as a missing entry. We also set up automatic push notifications that served as reminders for participants to enter data for each meal. These push notifications were programmed to be sent at predefined times (3 times daily) for breakfast, lunch, and dinner. Additional reminders were sent if entries were still not detected at the predefined times.

Besides allowing food/drink and portion size entries, Calci-app also contained a Web dashboard for researchers to view participants’ entries, including the calcium levels for each entry. Participants were provided with unique log-in identifiers and passwords to download and access Calci-app from Apple Inc’s official app store, iTunes, or the Google Play Store for Android operating systems.

### Beta Testing

Nowpos designed the graphic interface, functionalities, and navigation. We agreed on the final app interfaces after several iterations. Nowpos developed a beta version of Calci-app and this was tested on a convenience sample of 10 young women within the research department over 5 days. The participants were given a Web-based diary to complete on each day of the beta test. The 5 main themes in the Web-based diary were (1) layout of interface, (2) ease of navigation and entry, (3) frequency and content of reminders, (4) reasons for noncompliance, and (5) identification of technical flaws.

After reviewing and sorting the participants’ responses into themes, we identified the following main issues: (1) difficulty in learning to accomplish basic tasks (10 out of 10 participants), (2) time-consuming to perform basic tasks (9 out of 10), and (3) app design was overall unsatisfying (8 out of 10).

The responses were forwarded to Nowpos and improvements were made in the final version of the app. The main changes were as follows: (1) improvement in search functionality, (2) addition of a summary screen to view completed entries, (3) addition of a “recently added” list, (4) automatic synchronization of entries and backend database, and (5) removal of the window period to allow flexibility in entries. We found it particularly challenging to improve the sensitivity and intuition of the search function and numerous adjustments were required to eventually allow users to search with only keywords, instead of the exact description as specified in the database. The revised build also contained an automatic synchronization function to circumvent issues with poor Internet connectivity by storing entries and uploading data into the backend database when Internet connectivity was available. This function, together with the removal of the 48-hour data entry window, reduced the need for participants to manually submit their entries and prevented missing entries in the database in the event that participants forgot to click on the submit button.

The beta test participants also shared that the push notifications were excessive; therefore, we decided to send only 1 push notification at the end of each day. If no entries were detected, another notification was to be sent the next morning. Unfortunately, these changes required significant modifications to the underlying architecture of the app and would affect development cost and timeline. As such, we only adjusted the timing of the push notifications (to be sent 2 hours after mealtimes and reminders to be sent an hour later if no entries were detected for a particular mealtime).

To assist participants with downloading and navigating the app, we added information and instructions into the app: (1) Calci-app details, (2) instructions or getting started, (3) frequently asked questions, and (4) contact details. Following completion of the informed consent forms, participants were advised to log in to the app, read the information, and complete an assessment of understanding before starting day 1 of data collection.

**Figure 1:**
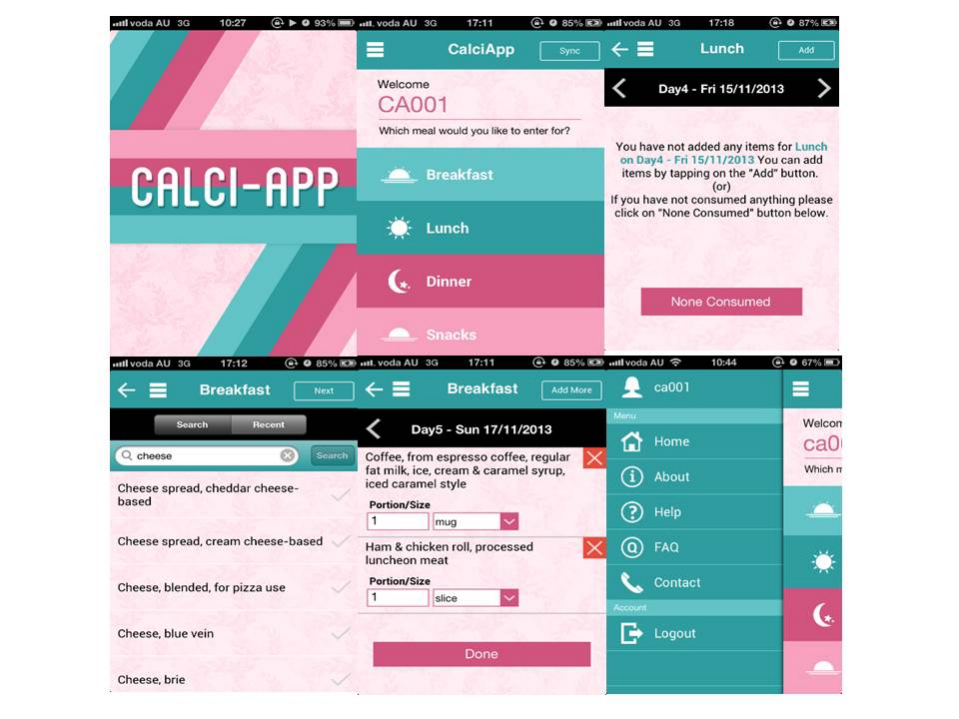
Screenshots of Calci-app interfaces.

The final version of Calci-app (see Figure 1) was released for feasibility and usability testing in the pilot study after adjustments from the beta testing were completed.

### Pilot Testing

#### Primary Outcome

We explored the level of compliance in young women with completing 5 days of food records in this pilot study.

A high level of compliance was defined as more than 3 days of completed food records and low-level compliance was 3 or fewer days of completed food records. A “completed food record” was one with all meals (breakfast, lunch, and dinner) entered, whereas a food record with either only 1 meal or 2 meals entered was considered a “partial food record.”

#### Secondary Outcome

We explored the acceptability of Calci-app as a mobile dietary calcium intake assessment and self-monitoring method. This was determined by using data collected from the usability questionnaire and mobile focus groups.

Participants for this study were recruited between October 14, 2013, and February 7, 2014, via telephone from the YFHI Launch study, which cross-recruited from previous studies—the YFHI Pilot [26] and Vaccine Against Cervical Cancer Impact and Effectiveness (VACCINE) studies [28]. Participants in these 2 studies responded to targeted advertisements on Facebook and were recruited through their expressions of interest via the study website.

Eligibility criteria for Calci-app were being female, 16-25 years old, resident in Victoria, Australia, and completed the YFHI study. Participants were excluded if they had current or past significant medical condition or conditions, if they were pregnant or breastfeeding, or if they did not own an iPhone or Android phone or were not willing to use them for the study.

Ethics approval was obtained from the Royal Women’s Hospital Human Research Ethics Committee (HREC 13/06).

Participants gave their written informed consent in the YFHI study, whereas for Calci-app participants gave their consent electronically. Informed verbal consent to participate was first obtained during telephone recruitment. Subsequently, an electronic copy of the HREC-approved informed consent form was sent to participants via LimeSurvey, an open-source survey tool developed by the University of Melbourne [20]. After receiving full information about the study, respondents provided and documented their consent to participate through LimeSurvey.

After logging in to Calci-app and reading the instructions, participants were asked to complete an assessment of understanding that comprised 5 questions (true or false). The aim of this assessment was to ensure that the participants understood when and how to accurately record their food and drink consumption.

Participants were asked to record their diet using Calci-app for 5 days over a 2-week period. The 5-day food record consisted of 3 nonconsecutive weekdays and 2 nonconsecutive weekend days to cover weekday versus weekend variations. Research has shown that a 3-day food record was sufficient to measure food intake [29]. For our study, we decided on a 5-day collection period to test the extent of compliance, while trying to limit participants’ fatigue.

All participants were to begin day 1 of app use on a Saturday and enter their records every alternate day until the fifth day, which fell on a Sunday. Participants were able to view the scheduled dates in Calci-app diary viewer once logged in. At the end of the 5-day food record, the researchers manually emailed the participants’ daily total calcium intakes to them.

### Usability Questionnaire

After participants completed the 5-day food record, they were asked to complete a usability questionnaire.

Quantitative feedback data about Calci-app was collected in the 5-item usability questionnaire using 5-point Likert scales (strongly agree to strongly disagree). The questionnaire focused on the following themes: (1) ease of use, (2) convenience, (3) intuitiveness, (4) time consumption, and (5) usefulness. The level of burden perceived by the participants was assessed on a scale of 0-10, with 0 being not troublesome at all.

### Mobile Focus Group

Completion of the 5-day food record and usability questionnaire was followed by an invitation to participate in a focus group. The topics covered during the interviews included evaluation of technology, integration and impact on everyday routine, acceptability and reasons for noncompliance, perceived impact on health, long-term implementation, and anticipated involvement and perception by others. These topics have been covered in other studies examining the use of mobile phone apps for health interventions [30-34]. The focus groups were conducted using a free mobile phone app called WhatsApp Messenger (Whatsapp Inc) [35] in a chat group created for this purpose.

### Statistical Analyses

Statistical analysis was performed with SPSS (IBM Corp, IBM SPSS Statistics for Windows, version 21.0). All quantitative data were analyzed using descriptive analysis and results are reported as n (%). Linear regression was used to assess participants’ compliance with food recording during the study time frame.

Thematic analysis was undertaken to analyze qualitative data. All analyses were performed using per-protocol approach.

## Results

**Overall Compliance**

On the basis of the eligibility criteria, 129 subjects were excluded (had eating disorder, did not own a mobile phone, were older than 25 years, and did not live in Victoria, Australia). Of the 90 young women who met the eligibility criteria, 54 (60%) agreed to participate. Ultimately, 40 participants completed the study. The other 14 participants withdrew consent (3 participants) and were lost to follow-up (11 participants).

**Figure 2:**
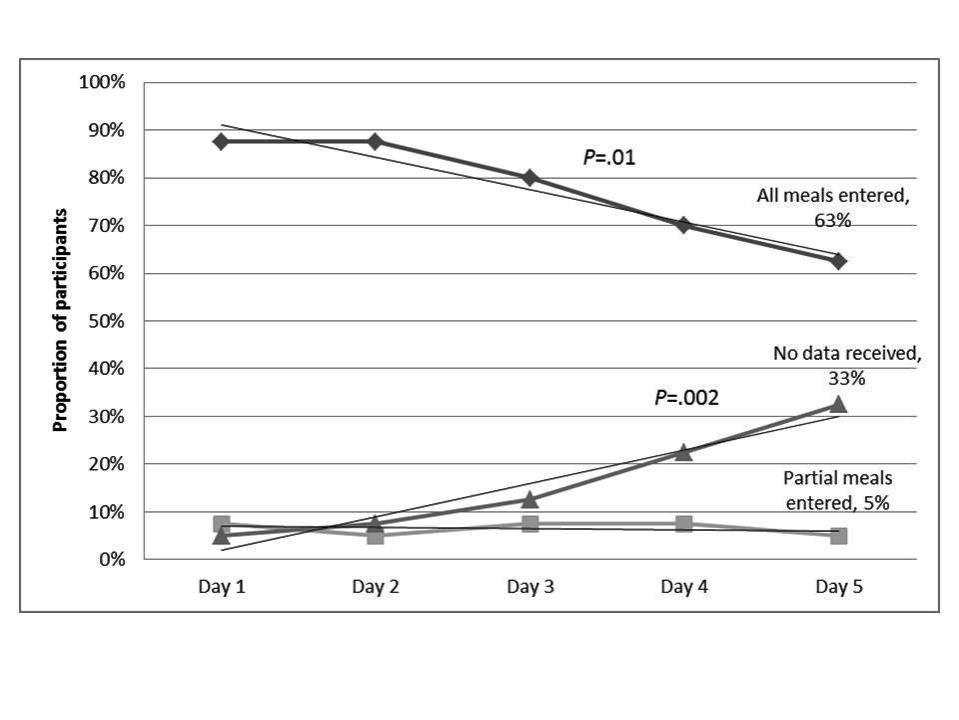
Compliance in meal entries over five days measured by proportion of participants (%) with completed food record, partial food record or no food record (n=40).

Overall, 78% (31/40) of study participants completed the 5-day food record with high compliance levels (defined as more than 3 days of full or partial completion). There was a significant reduction in the proportion of participants completing all meal entries over the 5 days (*P*=.01; see Figure 2) and an increase in the proportion of participants who did not enter any data at all over the 5 days (*P*=.002), especially from day 2 onward. Participants who entered partial meal entries (at least one meal entry) remained constant.

### Usability Questionnaire

**Figure 3:**
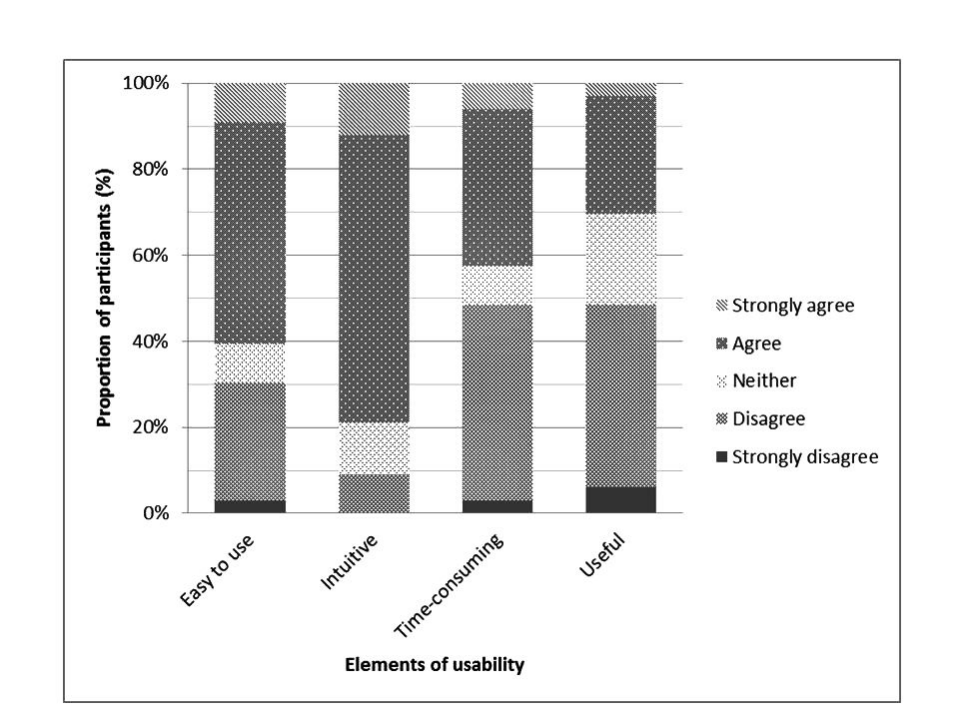
Responses to Calci-app usability questionnaire (n=33).

A total of 33 participants (83% of the 40 participants who completed the study) completed the usability questionnaire. Of the 33 participants, 20 respondents (61%) found Calci-app easy and convenient to use, 26 (79%) found the app design intuitive and not confusing to use, 14 (42%) found Calci-app time-consuming, and 10 (30%) found it useful (see Figure 3).

### Mobile Focus Group

A total of 22 participants participated in 5 focus group sessions between January 13, 2014, and February 21, 2014. Each focus group session had 3 to 8 participants and lasted for 30-40 minutes. Of 22 participants, 16 joined the focus group session within 1 month of completing day 5, and 6 of them had the session between 1-2 months after they completed day 5.

Thematic analysis of the focus group interviews aimed at understanding participants’ experiences with Calci-app revealed 5 distinct themes: (1) good aesthetics and ease of use promoted app usage, (2) frustration and time consumption reduced app usage, (3) ability to add accurate entries may improve app usage, (4) owning a purpose motivated app usage, and (5) diet concerns hindered app usage (see Table 1).

**Table 1:** Themes and codes derived from qualitative analysis of Calci-app mobile focus group interviews.

Theme	Codes
Good aesthetics and ease of use promoted app usage	Easy to pick up and use Preferred dietary assessment method Attractive design
Frustration and time consumption reduced app usage	Slow loading Unintuitive search function No directive interface Lack of database categorization
Ability to add accurate entries may improve app usage	Inaccurate search function Portion size cannot be estimated Portion size entry not simple and flexible Lack of extensive database with basic options
Owning a purpose motivated app usage	Knowing calcium level in food influences choices Want comparison between actual and target intake Potential for app to provide health education Want to see trend of past entries Possible data sharing with health professionals
Diet concerns hindered app usage	App usage heightened dietary awareness Concerns about diet may lead to eating disorders

#### Good Aesthetics and Ease of Use Promoted App Usage

The color scheme, graphics, layout, and fonts used in Calci-app were considered attractive to young women, and this created interest in using it. Several positive comments were made regarding the app design, interface (layout), and functions. The letters represent individual respondents.

F: *Designs were nice. Made me want to use it.*

L: *The app was nicely designed and I liked the colour scheme.*

M: *I found the interface really easy to navigate, logically set out and minimal buttons made it simple to use.*

Almost all participants appreciated the simplicity of learning how to use the app and the ease in its daily use. Participants also appreciated the use of a mobile phone food record as a method of dietary assessment.

V: *It is helpful being on your phone, which is always with you.*

S: *Easy to use :) instructions in the email were well written. No worries with navigation! Simple to “get around.”*

Q: *It was pretty easy- and I'm quite technologically challenged!*

#### Frustration and Time Consumption Reduced App Usage

Although the aesthetics of Calci-app and the ease of learning to navigate and use the app might have caught the participants’ attention, overall app usage was impeded by a poor and unintuitive search function that produced irrelevant hits.

I: *I found the app very easy to use. It was well designed and the menus made it easy to navigate. One problem was that some search terms (simple things such as raspberries) would return a lot of hits, which was frustrating.*

Q: *I found it easy to navigate but was a bit clumsy when I had to add individual ingredients to a meal and the items I'd actually typed in were at the bottom on the list but the loosely related brand specific items were at the top of the list.*

The participants suggested several improvements to increase usability. They suggested a more intuitive search function with the database categorized to prevent the need to add individual ingredients for a meal and to minimize the list of search hits. They also preferred common food items to appear on the top of the search list to remove the need to scroll and find their selection.

The addition of manual and automated functions would also improve the food entry process, for example, manual entries of food and recipes, bar code scanning, favorites list, and a longer recently added list.

Participants also found that the app was rather slow and it was not clear at times whether it was frozen or was loading. Through their comments, it was apparent that users may only have a short amount of time to enter food records and they may not be particularly patient with an app that is slow.

L: *I agree with the loading being slow at times. Sometimes it could take quite a while to enter a meal so it wouldn't be something that you could do all the time.*

I: *My issues weren't that bad! I'm just impatient and found waiting a minute or so annoying.*

C: *I ended up writing everything down and entering it at the end of the day because of the slow loading.*

#### Ability to Add Accurate Entries May Improve App Usage

The need for accurate food entries was a repeated theme among the young women and the inability to locate exact food items frustrated them. Furthermore, 3 participants expressed that they were on special diets (gluten-free, lactose-free) and could not find their products in the database.

T: *I had a lot of trouble finding relevant food. There seemed to be very few gluten free options and sometimes the items listed were so specific that it was hard to find just a basic food item.*

K: *I found it pretty easy to use and understand. However, due to my food allergies not all of the food items or brands I use were listed. So finding similar things was hard at times. So my info may not have been as accurate as it could have been.*

We further analyzed the participants’ comments and noted that, besides an unintuitive search function that might cause inaccurate entries, other database limitations were described. These were broken down into 3 main issues:

- Database was not sufficiently extensive
- Items were poorly described
- List of search results was too long and it strained users’ attention

Besides imprecise food selection, participants also disliked the inability to accurately estimate portion sizes. Although they found the function of selecting portion measurements simple and basic, they found it frustrating to guess the portion sizes. For example, a cup of milk is subjective and it was not possible for them to estimate the amount in milliliters.

E: *The search style took some getting used to. And the portions options were so inaccurate, how big is a “serve.”*

D: *And say I had tomyum soup, it was hard to choose from the list and a “bowl ” could mean a wide range of sizes.*

#### Owning a Purpose Motivated App Usage

When asked whether they could foresee young women using this app, most respondents agreed that only those who have a purpose for using it may use it long term. Examples may include the case of someone with bone problems or those who had been informed by their general practitioners (GPs) that they have low bone density. Many felt that it would be beneficial if the app had a data sharing function for GPs or other health professionals to view their calcium intakes and make recommendations. Many pointed out that they would not use the app if it was just a food diary.

M: *I think most would see it as unnecessary unless diagnosed with a bone problem, I don't think many of my friends would be interested in logging food, many would do so intermittently which almost renders the process useless.*

J: *I'd love my GP to recommend an app like this, or anything that's not just taking tablets.*

Almost all participants said that Calci-app increased their awareness of food selection and that they were encouraged to select food with higher calcium content. However, they would benefit more if Calci-app could present instant feedback on their total daily calcium intake against the recommended levels. Some wanted the app to go beyond a basic food diary and become an informative health intervention app that is able to suggest calcium-rich foods to the users, ring an alarm if calcium intake is low, as well as feature other young women’s calcium intake through the use of social media.

D: *Umm we weren't given any values so I was unaware about how much I was consuming but if it gave us a breakdown at say the end of each day that might reflect the choices I make for my next meal.*

F: *Well there isn't any immediate feedback on the actual intake. So it's impossible to know whether you are doing well for the day or not. It did make me more aware of what and when I was eating. I was thinking more whether what I was eating would have high calcium content.*

J: *I was curious of foods other than milk, cheese, cream and yoghurt had dairy, but I still don't really know. And how much others consume, like what's the average calcium intake for my age or bmi.*

U: *And a list of foods with high calcium if we're running low on inspiration.*

#### Diet Concerns Hindered App Usage

It was of interest that although the use of Calci-app was able to motivate positive food choices to increase calcium intake, the heightened awareness in their diet also led to negative thoughts and emotions, such as guilt and discomfort. A total of 6 participants highlighted a concern that the app may cause potential eating problems in young women, who are often vulnerable to issues with their body image.

R: *Like: it was convenient that I could record meals at any time during the day Dislike: keeping such a close eye on my diet as it made me feel guilty at times.*

S: *To be honest I didn't mind doing it for the study and I know I need more calcium and iron but I wouldn't be comfortable doing it long term.*

## Discussion

### Principal Findings

Here we described the process of developing Calci-app, designed for self-monitoring of dietary calcium intake in young women. Our goal in this pilot study was to test the usability of Calci-app and to determine the compliance of young women with Calci-app in recording their dietary intake before it is used in an interventional study. This study’s outcome reveals the barriers and facilitators in the use of Calci-app by young women to self-monitor dietary calcium intake to bring about an improvement in dietary consumption of calcium.

Our study showed relatively high compliance with dietary intake recording. However, the mobile self-monitoring method did not achieve high-level adherence beyond 3 days of recording. This finding is consistent with another study, which also reported an average of 3 days of dietary recording from participants in a free-living environment [36]. Another study also found low adherence regardless of whether self-monitoring was done on paper or PDA [23]. The compliance seen in our study may be a result of participants simply following the researcher’s advice. On the contrary, adherence is a long-term commitment where participants internalize and take ownership to act in order to produce a therapeutic outcome (ie, self-monitoring, self-therapy, etc) [37]. The use of mobile phone apps is often sporadic and transient [38], which was also echoed by the participants during the focus group. Hence, other strategies may need to be considered to motivate participants to adhere to the intervention.

A possible solution is to personalize the self-monitoring experience by enhancing participants’ connection to the intervention and improving support by minimizing negative experiences [39]. Even though participants found Calci-app easy and convenient to use, they described it as time-consuming and not useful to them. Providing a simple and intuitive search function may address the time consumption and technical obstacles that distracted them from using the app. However, it is difficult to instill a need or readiness for behavior change that will motivate young women to use Calci-app continuously. Participants could not identify a purpose for which to use Calci-app and this may be related to their perceived susceptibility to osteoporosis, which is a key component when assessing adherence to a behavioral intervention [40]. Research has shown that young adults perceive a lower susceptibility to osteoporosis [41,42] compared with older adults because osteoporosis is largely an older person’s disorder. Moreover, young women do not perceive osteoporosis as a severe problem, thus reducing their sense of urgency to protect themselves from the disease [41]. This barrier may be mitigated by adding a component of education about the potential consequences for bone health with their inaction when they are young. Providing individual feedback on their bone mineral density to individuals may also help to improve engagement [43], but it may be impractical on a large scale because it is relatively expensive and uncommon for young women to have bone mineral density testing.

Participants in our study also reiterated the need for immediate feedback on their actual total calcium consumption and as a comparison against the recommended daily intake levels. Although the technique of self-monitoring and visibility of the calcium content in each food item made them more aware of selecting food with higher calcium levels, knowing their actual consumption against the targeted levels may influence the choices they make at the next meal. Research has established that few entirely mobile phone app–based interventions have been effective. The greatest value is found when education and counseling are combined with feedback and ongoing self-monitoring [12]. This is most apparent in weight loss interventions, where research demonstrated that regular weighing with electronic graphical feedback prevented weight gain in young adults [44], and a Web-based intervention administered with caloric and weight feedback prevented weight gain in college students [44]. Additionally, a recent systematic review of mHealth interventions revealed that interventions that integrate a greater extent of behavior change techniques had the best outcomes [45]. A majority of the successful dietary interventions in young adults used behavior change techniques such as goal setting, personalized feedback, and advice depending on the phase of change that the participant was at [46,47]. However, details of the types of behavior change strategies most effective in our target population are yet to be determined.

It is noteworthy that although we can address the personal barriers in the uptake of Calci-app, such as purpose to use, time constraints, and the lack of motivation, we cannot remove the heightened awareness that dietary monitoring carries. Our study excluded young women with current or past eating disorders, yet our study participants shared that recording their diets made them feel guilty and uncomfortable. Weight management is a major determinant of eating habits [48] and dietary interventions may inadvertently promote eating disorders [47]. Therefore, intervention designs should include components to address body image issues and encourage body satisfaction.

One of the strengths of this study was the use of a mobile method for focus groups. To our knowledge, our study is the first to conduct focus groups using a social media platform (WhatsApp Messenger). Focus groups are traditionally conducted face-to-face and they must be performed at times and locations most suitable for participants. Participants may also feel uncomfortable sharing their opinions freely in a face-to-face group setting [49]. Additionally, focus group sessions are either audio- or video-recorded and the content transcribed by researchers, which can be tedious and time-consuming. One hour of audio recording is equivalent to 8 hours of transcription [49]. Furthermore, the quality of the voice recording is largely dependent on the capabilities of the microphone to capture voice volume variations of the participants and the ability to identify the voices of the speakers [50]. The use of mobile focus groups may overcome these issues. We conducted 5 focus groups in total and had 11 no-shows after excluding the participants who did not complete the usability questionnaire. This is equivalent to approximately 2 to 3 no-show attendees per focus group and it is comparable to previous research findings that suggested that each focus group will likely have 2 no-show attendees [51]. The name of the participant is keyed into WhatsApp Messenger’s user profile, which identifies the participant when she responds in the chat group. The focus group conversation is saved in the chat group and can be exported into a .txt file and emailed to the researcher for analysis. However, the mobile focus group is limited by its inability to assess participants’ body language and voice tones, and these critical cues therefore are not available in data analysis. We find that this may be moderated by probing and expanding each response to discuss the participants’ feelings associated with their opinions. Although we find that the mobile focus group similarly fosters the interactive nature of face-to-face focus group, the participants’ ability to share freely may be hindered by the limitation of texting long responses and explanations. Although we only used this social medial platform for mobile focus group, it is yet unknown whether the use of social media can improve effectiveness of interventions. These platforms are far-reaching and not expensive and can be useful tools to encourage content sharing and interactivity [46].

### Limitations

Limitations of this study are similar to other studies in self-monitoring for behavioral change [52]. The sample in this study consisted of mainly white young women, and a majority of them were university students. The participants were recruited from a sample of women who volunteered to participate in the YFHI study through an expression of interest. Although research has shown that educational level did not seem to influence nutrition intervention outcomes [53], there may be inherent characteristics in these volunteers that may lead to the observed outcome of relatively high compliance, such as interest in general health, higher awareness about health issues, and the importance of contributing to research. Therefore, it will be imperative to determine the uptake of Calci-app in a more diverse population living in the “real world” and over a longer time.

The attrition rate in the study was high with 7 participants who did not complete the usability questionnaire and an additional 11 participants who did not attend the focus groups. High attrition rates in eHealth and mHealth interventions are not uncommon [54,55], but the loss of valuable feedback from participants who drop out may have influenced the usability outcomes of the Calci-app that could inform future intervention designs.

### Conclusions

We presented a detailed description of the development process of Calci-app and an evaluation of its usability and acceptability to self-monitor dietary calcium intakes. The findings from this preliminary study demonstrated acceptable use of Calci-app to self-monitor calcium consumption. However, for regular and long-term use, the self-monitoring function in Calci-app could be expanded to allow participants to view their total daily calcium intake against the recommended daily intake. Additionally, to facilitate sustainable lifestyle behavior modifications, a combination of various behavior change techniques should be considered, such as education, goal setting, and advice to participants based on their stage of change. Other negative factors limiting adherence to app use such as time consumption, frustrating technical functions, and body image issues also need to be addressed. Although other interventions have used dietary mobile phone apps to improve various lifestyle outcomes, to our knowledge there is no research investigating the use of a mobile phone app to improve risky lifestyle behaviors in young women for better bone health. The feedback on barriers and facilitators from testing Calci-app will be used to design a bone health mHealth intervention to modify risky lifestyle behaviors in young women for better bone health outcomes.

## Abbreviations

GP: general practitioner
HREC: Human Research Ethics Committee
PDA: personal digital assistant
YFHI: Young Female Health Initiative
